# Integrated Environmental Health Impact Assessment for Risk Governance Purposes; Across What Do We Integrate?

**DOI:** 10.3390/ijerph13010071

**Published:** 2015-12-23

**Authors:** Erik Lebret

**Affiliations:** 1 National Institute of Public Health and the Environment—RIVM, P.O. Box 1, 3720 BA Bilthoven, The Netherlands; erik.lebret@rivm.nl; Tel.: +31-30-274-4194; 2 Institute of Risk Assessment Sciences—IRAS, Utrecht University, Yalelaan 2, 3584 CM Utrecht, The Netherlands

**Keywords:** integrated environmental health impact assessment, risk governance, environmental burden of disease, economic costs of environmental health impact, risk perception and acceptability, hierarchy of science, cognitive distance

## Abstract

Integrated Environmental Health Impact Assessment (IEHIA) can be considered as an element in the third phase of environmental risk management. Its focus is on providing inclusive descriptions of multiple impacts from multiple stressors in such a way that they can be evaluated against the potential societal benefits of the causes of the stressors. This paper emphasises some differences and difficulties in the integration across professional paradigms and scientific fields, across stakeholder perspectives and differences in impact indicators that emanate from these different fields and paradigms.

## 1. Introduction and Background

Integrated Environmental Health Impact Assessment (IEHIA) is a rather new concept that is receiving increasing attention in the 21st century. This narrative review describes some of the major challenges that are encountered in performing an IEHIA, in particular those that arise from the integration across professional paradigms and scientific fields, across stakeholder perspectives and differences in impact indicators. The work presented in this manuscript is partly grounded in the INTARESE (Integrated Assessment of Health Risks of Environmental Stressors in Europe) research project [1] under the EU 6th Framework programme and uses material from technical reports and deliverables from that project. The website [2], developed jointly from the sister-projects INTARESE and HEIMTSA (Health and Environment Integrated Methodology and Toolbox for Scenario Assessment) provides a guidance system, a toolkit, case studies, and background information for the performance of IEHIA . The more technical information to perform IEHIA can be found there.

Potential health effects of environmental stressors have since long been a cause for concern. Broadly, there are three main phases in the management of the health risks of environmental stressors (after [3]). In the first phase, going back to the Hygienist Movement in the 18th century, environmental risk management effort was directed to reduction and removal of the easily *observable* pollutants, e.g., open sewers, smoke plumes, algal blooms, foaming surface water with floating dead fish, and visible or malodorous soil pollution. Also clearly discernible peaks or clusters in mortality or morbidity, e.g., following smog episodes in the Meuse Valley (1938) [4], the London Smog (1952) [5] or Minamata mercury poisoning in the 1950’s and the decline in bird populations due to DDT *i.e.*, “Silent Spring” triggered debate, research and policy actions.

**Figure 1:**
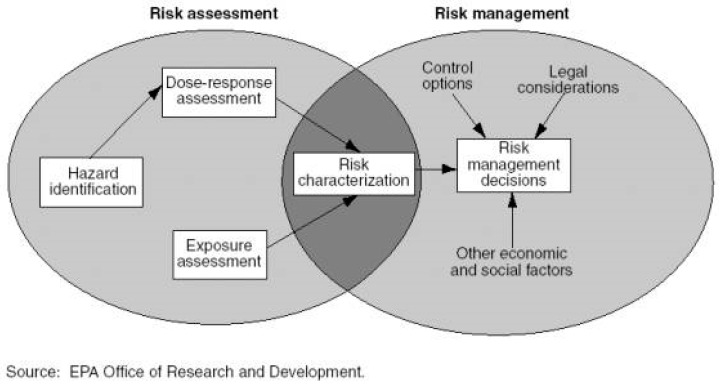
Diagram of the structural framework of the National Research Council (NRC) risk assessment/risk management paradigm [8].

The second phase focused on management of *measurable* pollutants, such as toxic chemical substances, radiation or noise measured in environmental media or as biomarkers in (human) bodily materials. This phase emerged in the second half of the 20th century, where technical developments allowed the measurement of pollutants at increasingly lower levels in a variety of (environmental) media. Parallel to development of extensive environmental monitoring, a set of environmental quality standards was developed and formal risk assessment procedures were put into place, e.g., the Dutch Policy Report Environmental Quality Standards in 1976 [6]. In that period, the USA National Research Council’s “Redbook” Risk Assessment in the Federal Government: Managing the Process [7] has dominated thinking about risk assessment since its publication. It discerns four steps in risk assessment, (1) hazard identification; (2) dose-response assessment; (3) exposure assessment; and (4) risk characterization (Figure 1).

Hazard refers to the property of an environmental stressor to do harm, *i.e.*, is refers to its intrinsic properties that elicit a biological response and that can result in adverse health effects. Risk is typically seen as a function of (i) the probability of an uncertain, but negative (undesirable) outcome (e.g., an adverse health effect) and (ii) the nature (e.g., mortality, disease, reversible physiological effect) and magnitude (amount of people involved) of the outcome.

Environmental quality standards were developed for specific stressors, sometimes for groups of substances with similar working mechanisms, e.g., dioxins, Polychlorinated Biphenyls (PCB’s) or Polycyclic aromatic hydrocarbons (PAH’s). These environmental quality standards, together with emission controls, regulations, and compliance monitoring have substantially reduced emissions of environmental stressors to the environment and improved public health.

In the late 1980s, social science publications about risk and risk perception emerged, e.g., [9,10,11]. Risk was not only defined as a function of probability and number of people affected, but as a social construct, where psychological aspects as well as social and cultural context together define the acceptability of the risk. The awareness that risk was more than a number [12] gradually led to the understanding that traditional concepts of quality standards, “unit risks” and “maximum tolerable risk” benchmarks were insufficient to govern risks. Other aspects, like involuntary nature of the risk, unobservability and uncontrollability of the risk, catastrophic potential, and inequity in distribution of risks and benefits were recognised as key elements in the evaluation of the acceptability of risks. This led to the search for alternative, more inclusive approaches, which involved among other things, stakeholder dialogues and risk-benefit considerations.

These more inclusive approaches can be described as the third phase of modern day environmental risk management, where the efforts are more directed toward striking a balance between the adverse risks and the societal benefits of the polluting activities. The focus is no longer exclusively on the direct hazards of specific environmental stressors, but is also focused on more diffuse, indirect and long-term problems, often acting at the international or global scale [13]. Risk perceptions and concerns, equity aspects, risk-benefit considerations, sustainability, uncertainty and precaution have become part of the risk appraisal and risk discourse. This also implies that not all risk problems can be considered similar in nature and therefore cannot be treated in the same way; tailor-made approaches are needed. The process of assessing and managing risk in a more inclusive way is often referred to as “risk governance”. The Whitepaper on Risk Governance; Towards an Integrative Approach from the International Risk Governance Council [14] provided such an approach and stimulated thinking about risk governance. In particular, risk governance differentiates between different types of risk problems with varying degrees of complexity, uncertainty and ambiguity (and combinations thereof), and proposes differentiated risk management strategies, instruments and stakeholder participation, commensurate to the complexity, uncertainty and ambiguity of the risk problem. The evolution from the risk assessment/risk management paradigm toward risk governance can be observed from the series of reports from for example the USA National Risk Council on Risk Assessment [15,16,17], the Dutch Health Council [12,18,19], the Joint Environment and Human Health Programme of the UK Research Councils [20], parallel reporting from other countries, and in the primary literature. Integrated environmental health impact assessment is an important element in modern risk governance. 

## 2. What Does Integrated Environmental Health Impact Assessment Entail?

### 2.1. Integration of What Exactly?

**Figure 2:**
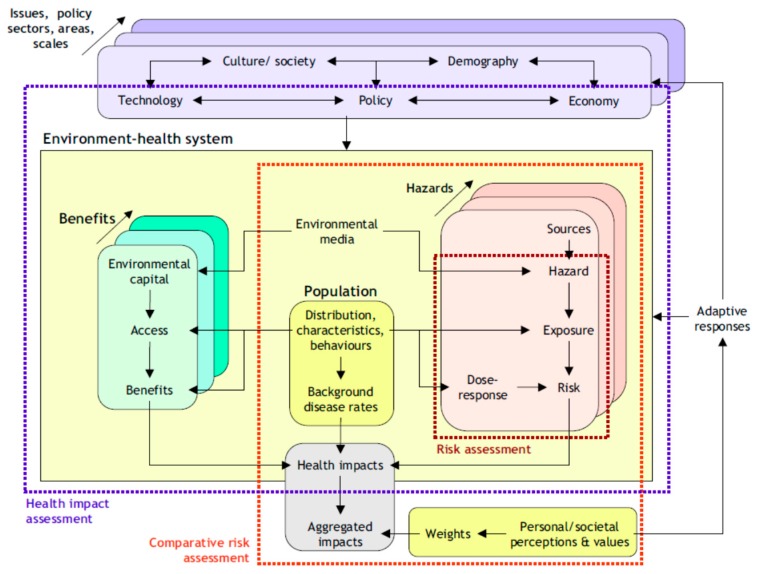
Structural framework of Integrated Environmental Health Impact Assessment (IEHIA) in relation to other forms of risk and impact assessment (from [21]).

IEHIA combines elements and approaches from frameworks of assessments from different fields. As Briggs pointed out, it draws from risk assessment, environmental impact assessment, health impact assessment and comparative risk assessment [21] (*cf.* Figure 2). All these fields use their own definitions and terminology, and differ in context, legal foundation, and action orientation. Impact assessments are typically oriented towards the evaluation of changes due to the implementation of specific projects or policies; they often have a legal basis. The element of “integration” is also interpreted in a variety ways. It is used to indicate integration of exposures across various media and uptake routes (e.g., as in integrated exposure to metals, pesticides, or dioxins), integration across the causal pathways from source to health effects (as in DPSEEA Driving force, Pressure, State, Exposure, Effect and Action [22]), integration of exposure to mixtures (e.g., endocrine disrupting chemicals), integration across risks and impacts of environmental stressors coming from the same source or economic or social activity (e.g., impact of fossil-fuel based traffic comprises health impacts from air pollution, noise, traffic accidents, energy depletion, greenhouse gas emissions, obesity, and health risks due to lack of physical activities, *etc.*), integration of diverse health effects (e.g., environmental burden of disease in Disability-Adjusted Life Years—DALY’s, an indicator that combines and aggregates loss of life years and loss of healthy life years due to environmental exposures [23,24]), integration of effects in humans and in ecosystems, or integration across scientific disciplines (as in interdisciplinary), or integration of various stakeholders’ perspectives in the assessment process (as in transdisciplinary). See Briggs 2008 [21] for some of these definitions as used in the “pedigree assessment fields” of IEHIA. Usually, the term “integrated” does not reflect the simultaneous integration across all such perspectives; typically it is restricted to only one or a few aspects of integration.

Briggs defined IEHIA as “A means of assessing health-related problems deriving from the environment, and health-related impacts of policies and other interventions that affect the environment, in ways that take account of the complexities, interdependencies and uncertainties of the real world”.

While this description still leaves much to the imagination, it does allow one to also consider (health) benefits associated with environmental changes. As with the description of the process of risk governance in the IRGC risk governance framework [14], the specific content of the IEHIA is defined in the first two phase of the assessment process, *i.e.*, the problem definition or issue framing and design phase [21]. Common elements in IEHIA are integration across the causal chain and integration of a variety of adverse outcomes in one or more aggregated indices.

### 2.2. Integration across Paradigms and Frameworks

**Figure 3:**
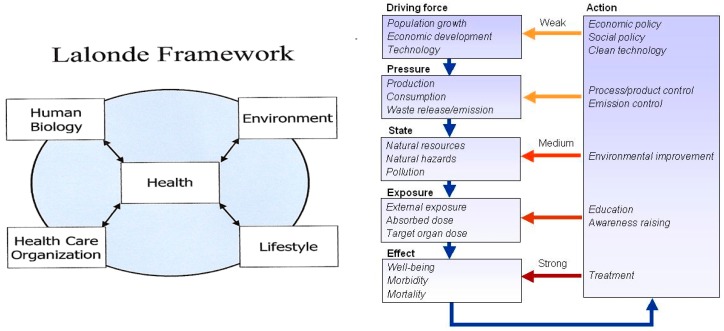
Common public health framework (left from [25]) and environmental sciences DPSEEA (Driving force, Pressure, State, Exposure, Effect and Action) causal chain framework (right, after [22] from [2]).

Given the pedigree of IEHIA, the possible paradigms and frameworks abound and such paradigms and frameworks rarely cross scientific fields or branches. While the Lalonde model of health determinants [25] is easily understood among public health experts, the risk assessment paradigm (*cf.* Figure 1) is largely unknown in these circles. Conversely, the Lalonde model is hardly used in environmental sciences, where e.g., the DPSEEA framework and risk assessment/risk management paradigm are common (Figure 3).

**Figure 4:**
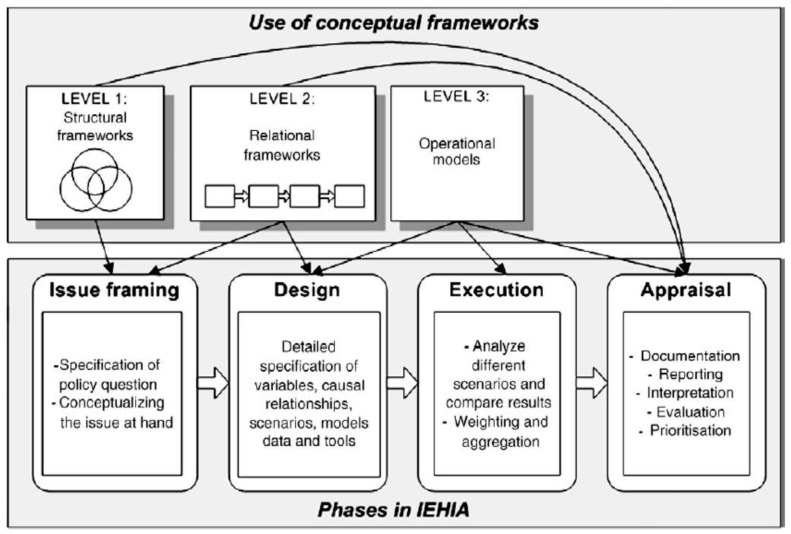
Phases in the process of an Integrated Environmental Health Impact Assessment and associated use of conceptual frameworks [26].

Knol and co-workers proposed a taxonomy for this variety of IEHIA-relevant frameworks and categorised them into structural, relational and operational frameworks. Figure 4 shows the role and place of these types of frameworks in relation to the four different phases of the performance of an IEHIA [26].

**Figure 5:**
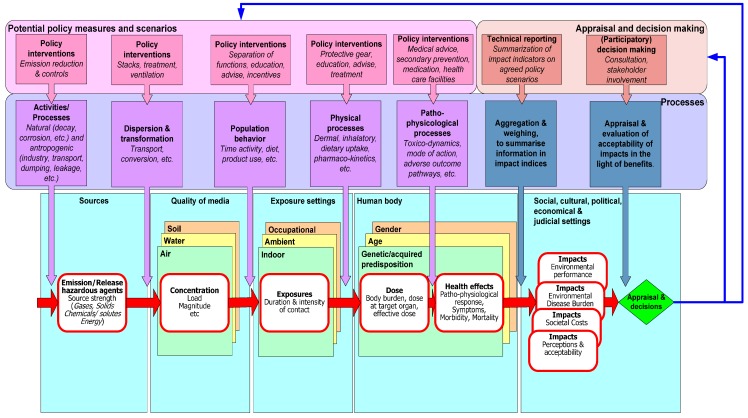
Causal chain, governing physical, chemical and biological processes and evaluation of acceptability of relevant impacts in relation to intervention options within the legal, social, economic, and cultural settings.

These frameworks are not mutually exclusive. In fact, many of the frameworks can be combined and nested, from structural to relational and operational. For example, the risk assessment model from Figure 1 also is an element in the risk governance framework and the IEHIA framework, possibly combined with the DPSEEA causal chain framework (*cf.* Figure 5). The execution of an IEHIA therefore requires understanding of the key elements of applicable frameworks (originating from different fields) as well as the specialised operational knowledge of the key physical, chemical and biological processes along the causal pathway from source to effect, together with an understanding of the specific contextual information about e.g., legal and cultural aspects of exposure setting. The figure illustrates not only the causal mechanisms, but also depicts the different contexts in which exposure may lead to adverse health impacts. For example indoor, occupational, ambient environments have different sets of exposure processes, different subpopulations exposed and quite different regulations and quality standards to govern the quality of these environments. It also shows the processes that rule transitions across the causal chain, together with possible policy scenarios to intervene and change these processes.

One should realize that for each arrow in the causal pathway, specialized expertise is needed to describe, understand and model these processes. For example to derive “dose to the target organ” from exposure in an environment, knowledge of actual exposure, Absorption/uptake, Distribution in the body across tissues, and Metabolic transformation and Excretion (ADME), is needed. These ADME processes are often described in Physiologically Based Pharmacokinetic or PBPK-models. The expertise for PBPK models is quite different in nature from the expertise to model the distribution of ambient air pollution from sources to recipients, which requires meteorological information and insight into chemical and physical transformation processes, as well as knowledge about behaviour of people as they move from one microenvironment to the next in their daily activities. Yet another group of experts would be involved in determining the overall health impact. Depending on the problem definition or issue framing, impacts can be described in regulatory terms (e.g., compliance or violation of emission guidelines, environmental quality guidelines, maximum tolerable daily uptake), or biological quality guidelines (such as blood lead levels); such a set would be informative for environment regulators. For a health impact assessment such a set would likely be complemented with indicators of health risks or impacts, e.g., mortality or morbidity indices. This latter set would be more informative for public health officials. IEHIA with integration across the causal chain, therefore, implies strong interdisciplinary interaction and cooperation.

### 2.3. Impact Indicators

There are numerous publications about environmental health impact indicators. Knol has summarized and evaluated some of these in light of their usefulness in IEHIA [27]. Three main groups of impact indicators are commonly used in IEHIA: (i) indicators that describe the environmental policy performance (EPI), *i.e.*, compliance with emission and quality standards, regulations and policy objectives [28,29]; (ii) burden of disease measures, e.g., Disability-Adjusted Life-Years, a measure that summarizes healthy life-years and life-years lived with disease, weighting for severity of the disease/disability [30]; and (iii) a monetary measure that summarizes the adverse health impact in terms of money [31]. These three groups of summary indicators are typically tied to the paradigms and frameworks in their respective fields. The EPI indicators draw from concepts from environmental sciences, the environmental burden of disease DALY stems from public health concepts, and the monetization of impacts originates from economics. The DALY uses severity weights to take into account the extent to which a disease affects quality of life of the patient. These are primarily based on (normative) judgments by physicians. The severity weights do not take into account to what extent the disease affects the patient’s family, work, or social network. In contrast, the economists use the concept of the normative willingness to pay (WTP) to weight the severity of health impacts. The WTP represents the supposed market value people are willing to pay to avoid the health effects. An often used metric of the WTP is the VSL, the value of a statistical life, the cost to society of one premature death. The WTP concept, at least conceptually, can take into account not only the severity of disease to the patient, but also to the patient’s family and social network, the cost of illness in terms of medical treatment, loss of labour and even altruistic aspects, where public goods like “clean air” are concerned. A common normative concept in monetary impact indicators is “discounting”. This implies that an amount of money at a given moment (say 100 €) has more value now, than the same amount in the future (say 10 years from now). This economic concept is usually not taken into account when estimating burden of disease indicators. While there are clear conceptual differences between DALY and WTP indicators, they do in fact require common input of information. Both DALY and monetary estimates require the estimation of the number of affected people (attributable numbers of mortality or morbidity) under the alternative scenarios. In IEHIA where both summary indicators are needed, care should be taken that the estimates are based on the same assumptions about attributable numbers. In other words, the teams of experts that make the DALY estimates should closely cooperate with the teams that make the monetary estimates.

Interestingly, while there is a wide literature from the social sciences about risk perceptions, as well as the concerns and acceptability of risk, there is no broadly accepted summary index for the degree of “acceptability” of risk for use in IEHIA, to our knowledge. Neither is the knowledge from social sciences on acceptability of risks captured in the DALY or WTP values used in health impact assessment. From the social science perspective regarding the appreciation of natural *versus* manmade risks as well as voluntary *versus* involuntary risks, one would expect that an asthma attack from involuntary exposure to manmade traffic-related air pollution would be less acceptable than one brought on by exposure to natural pollen, or other natural triggers or “acts of god”. Neither the DALY nor the economic valuation for e.g., “cost of illness” takes this into consideration. Istamto and colleagues in their international five-country study on WTP to avoid health effects of traffic-related air pollution and noise demonstrated that indeed risk perceptions and environmental concerns affected WTP stated by respondents, even to the degree that countries with relative high average WTP values, dropped in rank when their high risk perceptions and concerns were taken into account [32,33]. 

**Figure 6:**
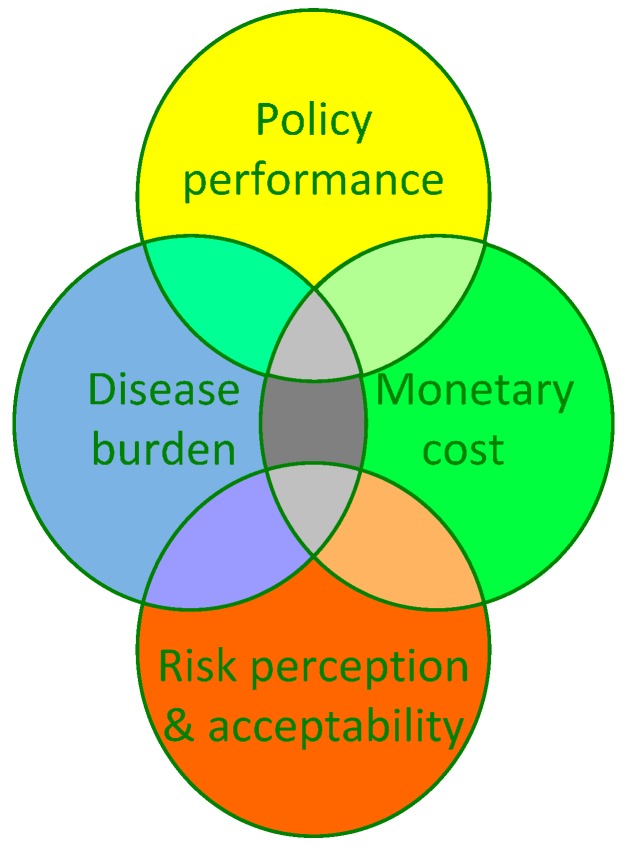
Differences and commonalities in (aggregate) impact indicators from different perspectives relevant to Integrated Environmental Health Impact Assessment.

An important lesson from these observations is that the normative choice of aggregate indicators in an IEHIA matters. While EPI provides a legal/regulatory perspective, DALY and WTP give utilitarian perspectives that include normative elements grounded in the paradigms and concept of their respective fields of science. Distributional issues like “social justice” which are concerned with the distribution of risks in the population *versus* the distribution of benefits from the activities that cause the risk, require other metrics and indicators when these issues are of concern in the assessment. As Figure 6 illustrates, the different perspectives from EPI, burden of disease, monetary valuation or perception of risks share some common elements, indicated as grey areas in the diagram (such as the number of people affected and the severity of effects). There are however many different features that are specific to only one or just two perspectives, indicated by non-grey areas.

While there is no summary index for the degree of “acceptability” of risk to date, and DALY and WTP indicators typically do not (explicitly) take perceptions and concerns into account, there are, however, approaches such as multi criteria analysis that allow one to combine risk perception aspects and to weigh these against e.g., EPI’s, DALY’s, or monetary costs. Thus, strong interdisciplinary interaction and cooperation is essential when performing an IEHIA because of the need of integration across multiple dimensions of impact.

### 2.4. Integration across Disciplines

**Figure 7:**
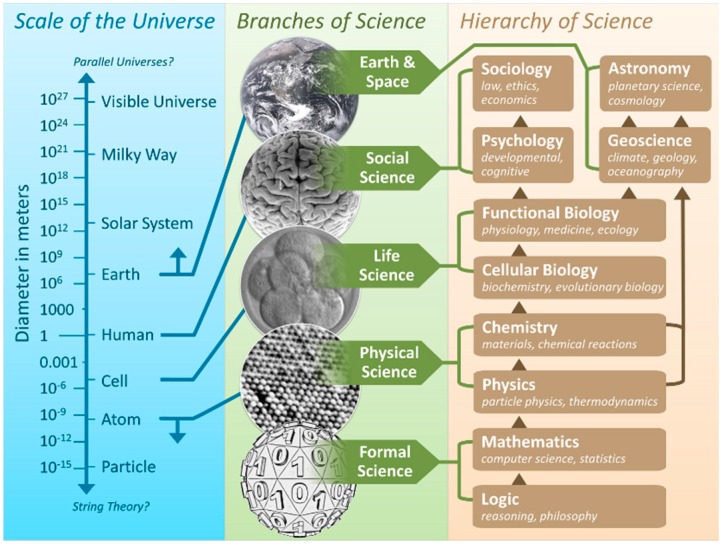
Branches of science and the hierarchy of science, mapped to the scale of the universe (from [35]).

The sciences typically applied in IEHIA are dominated by physical and life sciences; IEHIA also draws heavily on mathematics for modelling and statistics. With increasing globalisation, climate change and sustainability, geo-engineering and geosciences gain in prominence. With “concern assessment”, risk perception and communication as elements in risk governance, social sciences are increasingly included in IEHIA. The question than is “How do all these disciplines interact and relate in IEHIA?” Relationships (and hierarchy) of sciences have been an issue of debate going back to the 19th century [34]. One way of describing the relation between these branches of science, and the scale of the universe, is given in Figure 7. This description has the benefit that it indicates at what level risks and impacts as described by disciplines, manifest themselves. 

Regardless of any hierarchy, for IEHIA to be successful, the issue lies in the ability of experts from different disciplines to understand each other and to work together productively.

**Figure 8:**
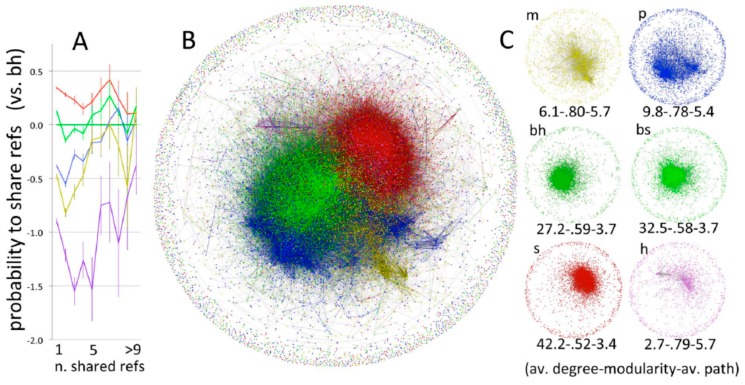
Maps of science of from Fanelli and Glänzel [34] showing probability of sharing references (**A**); network of shared references (**B**) and networks by domain (**C**). Bibliographic coupling network of papers, partitioned by scientific domain (total *N* = 28,477; yellow = mathematics; blue = physical sciences; darker green = biological-hard sc.; lighter green = biological-soft sc.; red = social sc.; purple = humanities).

**Figure 9:**
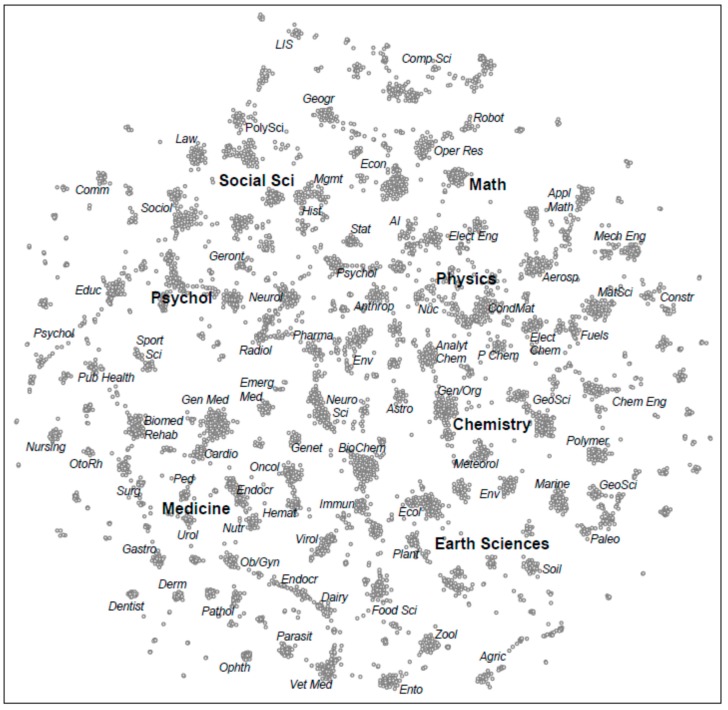
Maps of science showing clusters of journal-journal relatedness for major areas of science. Smaller labels denote the disciplinary topics of nearby large clusters of journals. (From [36]; reprinted by permission of Springer).

Bibliometric studies as from Fanelli D and Glänzel (Figure 8 [34]), or from Boyack and colleagues (Figure 9 [36]) clearly demonstrate the limited interaction between scientific disciplines.

**Figure 10:**
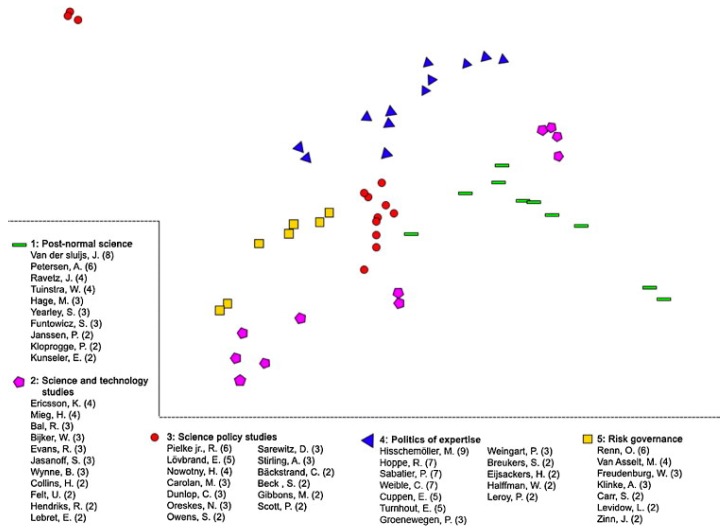
Co-citation analysis of reviewed literature on roles of scientists as policy advisors by different fields of science. (From [37]; reprinted by permission of Elsevier).

Moreover, the limited interaction is not restricted to interaction between broad disciplines, but is also manifest in different “schools of thought” on more defined and narrower topics. Spruijt and colleagues in their literature review of roles of scientist as policy advisors found distinct citation communities by discipline and field (Figure 10 [37]). Within citation communities, authors primarily refer to publications within their “school of thought” with few references to pertinent publications in other areas, compounding the issue of limited interaction between different fields of science.

**Figure 11:**
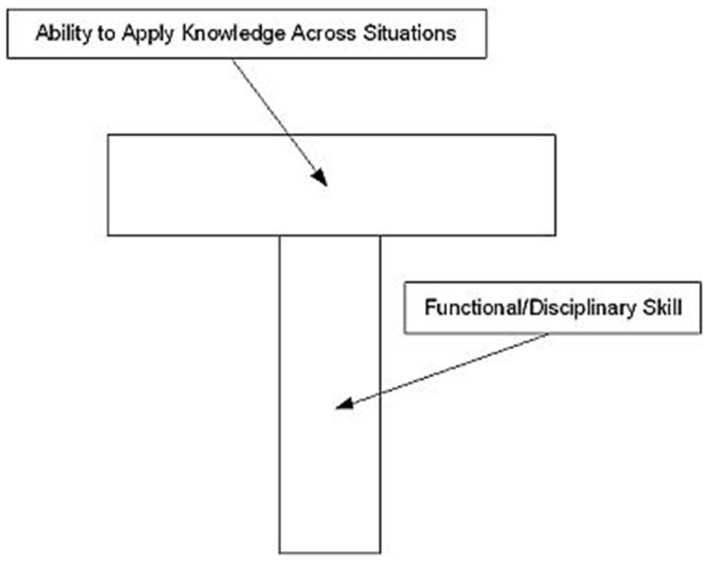
T-shaped skills of professionals [40].

As Fanelli and Glänzel put it “In an ideal science, scholars share a common background of established theories, facts and methods. This allows them to agree (usually after debate and further evidence) on the validity and significance of a new research finding, making it the basis for further theorizing and research… ”. Clearly, the lack of interaction between fields and the differences in scientific paradigms hamper such universal thinking and thus hamper interdisciplinary cooperation. In contrast to this “ideal science” Cohen and Levinthal argue that different people see, interpret, and evaluate the world differently to the extent that they have developed in different social and physical surroundings and have not interacted with each other [38]. In other words, environment and past experience determine ability to interact. This is particularly true for experts who have received years of formal extensive training and education in a particular field. They see, interpret and evaluate scientific information from their professional paradigms and conceptual frameworks. Anyone who has worked in teams with colleagues from very different branches of science will recognise these differences. They can be rewarding and frustrating at the same time. Nooteboom introduced the concept of an “optimal cognitive distance” for teams to productively cooperate toward a common goal [39]; in our case to perform an informative IEHIA. Paraphrasing Nooteboom one could argue that to ensure productive interdisciplinary cooperation in IEHIA, a trade-off needs to be made between cognitive *distance*, for the sake of effectively using knowledge from different experts, and cognitive *proximity*, for the sake of efficient absorption of knowledge across causal pathways, across disciplines, and across professional paradigms and frameworks. In other words, teams of experts should be composed of people who have the “cognitive flexibility” to communicate with experts from different fields and the ability to absorb their knowledge. These may not always be the top specialist experts. Alternatively, generalist experts are needed in the team to bridge the cognitive distance between team members. Members of an interdisciplinary team developing an IEHIA need what Leonard-Barton referred to as T-shaped skills or T-shaped professionals (Figure 11 [40]). It refers to (specialized) disciplinary and functional skills in the vertical of the T-shape and the additional skills, the horizontal bar in the T, the skills or abilities needed to apply knowledge across situations, a feature essential to interdisciplinary cooperation in IEHIA.

### 2.5. Integration across Stakeholder Perspectives

The concept of cognitive distance of course also applies to interactions between different stakeholders with regard to their perspectives in IEHIA, even more so than between experts from different disciplines. Different stakeholders apply different heuristics, argumentation, and (formal) decisions rules as well as holding different worldviews. Moreover, different stakeholders typically have different interests, even when cognitive distance would not be an issue. In IEHIA, failure to take stakeholder perspectives into account may lead to what are sometimes called Type IV errors. Type IV error refers to situation where the answer may in itself be correct and for the right reasons, but the answer does not address the question of concern. This may be the case where an IEHIA uses “utilitarian” impact indicators such as DALY or monetary values for health impact, while pertinent stakeholder questions are about social justice, the involuntary nature or uncontrollability of the risks, the uncertainty of risks, or procedural (in) justice. Stating that a risk (defined as a function of probability and nature of effect) is below the benchmark of maximum tolerable risk. e.g., risk of dying is below 10^−6^ per year is almost meaningless from the perspective of risk as a social construct, even more so where innumeracy in part of the audience is of concern.

## 3. Discussion

Integration of information to inform an environmental health impact assessment may take different forms, across multiple stressors from the same source, across different impacts from the same stressor (e.g., acute and chronic respiratory and cardiovascular morbidity, and lung cancer and premature mortality of air pollutants), across multiple exposure pathways of a stressor, or across mixtures of substances with similar modes of action. Technical approaches and procedures are well documented in the literature and the web (e.g., website of the INTARESE/HEIMTSA projects, or the Finnish online portal OPASNET (http://en.opasnet.org/w/). While the technical elements are challenging, aspects of the assessment process and the required inter- and transdisciplinary cooperation are often disregarded. The challenges already formulated in 1996 by the National Research Council are equally relevant for IEHIA:

1. Getting the science right
2. Getting the right science
3. Getting the right participation
4. Getting the participation right
5. Developing an accurate, balanced, and informative synthesis

Experts usually stress the first aspect; they are familiar with the debate about getting the science right. The other elements form bigger challenges; which fields of science should be involved, which stakeholders, and how to organise the participation process. Most importantly, how to avoid type IV errors and how to get to informative synthesis of information that allows informed decision making, doing justice to the diverse perspectives from the regulatory contexts, the different fields of science, and from different stakeholders. Almost two decades following the 1996 NRC report, these challenges remain of crucial importance. Only a few worked examples can be found in the literature, for two main, and related, reasons: (i) responsible authorities seem reluctant to commission and engage in IEHIA’s; and (ii) limitations in (organisational) competence. The first reason may be gradually changing with risk governance gaining in prominence as exemplified by Grenelle de l’environnement in France [41] or the “Explicitly dealing with safety” approach in the Netherlands [42]. Limitations of competence to perform IEHIA are in part due to the lack of investment. As the National Academy of Sciences puts it [15] “Each organization responsible for making risk decisions should work to build organizational capability….”. This of course will only happen when IEHIAs are indeed commissioned. But there are deeper underlying problems with respect to the capabilities to perform IEHIAs, some of which are mentioned in the sections above. There is a serious lack of interaction between different fields of science pertinent to IEHIA. Academic curricula and training provide little opportunity to engage in real interdisciplinary work. Funding programmes for interdisciplinary research in IEHIA remain rare, and interdisciplinary work appears more difficult to get published. Scientific recognition mainly originates from within defined mainstream professional fields. Some other institutional and cultural roadblocks for convergence of knowledge from different disciplines are described in the literature e.g., [43].

In addition, technological developments in many branches of science have led to vast advances in knowledge in recent years and led to further (hyper)specialisation (e.g., toxico-genomics, proteomics, metabonomics, biostatistics, “lab-on-a-chip”, “big data” and data mining, miniaturisation of sensors, smartphone-based sensors, *etc.*). Many of these developments are pertinent to IEHIA and may compound the problems of cognitive distance between fields, limit convergence and increase the need for the T-shaped professional to adequately perform IEHIA. Ironically, the “integration” for IEHIA will not come from the “I” shape which stands for the specialists’ knowledge, but requires the inclusion of “T-shape skills.

In this third phase of the management of environmental risks, IEHIA will be of increasing importance to modern risk governance. Great challenges remain in the technical conduct of IEHIA: integration across causal pathways, evaluation of mixtures of substances, informative description of uncertainties. Many normative issues await discussion and practical resolution, e.g., about whether and how to include environmental health impacts of hypertension, sleep disturbance or annoyance into burden of disease or cost of illness estimates. Dealing with cognitive distance and enabling convergence of knowledge across disciplines and across stakeholders perspectives will prove to be one of the biggest challenges in truly integrated environmental health impact assessments.

## 4. Conclusions

Technical elements in IEHIA are challenging and require in-depth understanding of biological, chemical, and physical processes in the causal chain from source to effect. At least as challenging are the assessment process and the required inter- and transdisciplinary cooperation to perform an IEHIA. Different and sometimes conflicting paradigms and legal, institutional, and cultural roadblocks hamper convergence of knowledge from different disciplines and can obstruct informative synthesis in IEHIA. There is a strong need to further develop organizational capability to perform IEHIAs. This involves among other things, funding of translational research, inclusions of T-shaped skills in inter-disciplinary teams and broadening of academic curricula to allow students and scholars to learn from different fields of knowledge.

## References

[1] Integrated Assessment of Health Risks of Environmental Stressors in Europe. http://www.intarese.org.

[2] INTARESE & HEIMTSA Guidebook. http://www.integrated-assessment.eu/guidebook/concept.

[3] Goldstein B.D. Personal communication.

[4] Firket J. (1936). Fog Along the Meuse Valley.

[5] Bell M.L., Davis D.L., Fletcher T. (2004). A retrospective assessment of mortality from the London smog episode of 1952: The role of influenza and pollution. Environ Health Perspect..

[6] (1977). Nota Milieuhygiënische Normen (in Dutch).

[7] National Research Council (1983). Risk Assessment in the Federal Government: Managing the Process.

[8] NRC Risk Assessment Paradigm.

[9] Slovic P. (1987). Perception of risk. Science.

[10] National Research Council (1989). Improving Risk Communication.

[11] Kasperson R.E. (1988). The social amplification of risk a conceptual framework. Risk Anal..

[12] (1996). Risk is More than Just a Number; Reflections on the Development of the Environmental Risk Management Approach.

[13] McMichael A.J. (1994). Global environmental change and human health: New challenges to scientist and policy-maker. J. Public Health Policy.

[14] Renn O., Graham P. (2005). White Paper on Risk Governance Towards an Integrative Approach.

[15] Stern P.C., Fineberg H.V., National Research Council (2005). Understanding Risk: Informing Decisions in a Democratic Society.

[16] National Research Council (2009). Science and Decisions: Advancing Risk Assessment.

[17] National Research Council (2012). Sustainability and the U.S. EPA.

[18] Health Council of the Netherlands (2005). Not all Risks Are Equal; Commentary on 'Premises for Environmental Risk Management.

[19] Health Council of the Netherlands (2008). Prudent Precaution.

[20] Moore M., Kempton P.D. (2009). A synopsis of the joint environment and human health programme in the UK. Environ. Health.

[21] Briggs D.J. (2008). A framework for integrated environmental health impact assessment of systemic risks. Environ. Health.

[22] Corvalan C., Briggs D.J., Kjellstrom T. (1996). Development of Environmental Health Indicators, Linkage Methods for Environment and Health Analysis, General Guidelines.

[23] De Hollander A.E.M., Melse J.M., Lebret E., Kramers P.G.N. (1999). An aggregate public health indicator to represent the impact of multiple environmental exposures. Epidemiology.

[24] Prüss A., Corvalán C.F., Pastides H., De Hollander A.E. (2001). Methodologic considerations in estimating burden of disease from environmental risk factors at national and global levels. Int. J. Occup. Environ. Health.

[25] Lalonde M. (1974). A conceptual framework for health. RNAO News.

[26] Knol A.B., Briggs D.J., Lebret E. (2010). Assessment of complex environmental health problems: Framing the structures and structuring the frameworks. Sci. Total Environ..

[27] Knol A.B. (2010). Health and the Environment: Assessing the Impacts, Addressing the Uncertainties. Ph.D. Thesis.

[28] Segnestam L. (1999). Environmental Performance Indicators: A Second Edition Note.

[29] European Environment Agency (2005). EEA Core Set of Indicators.

[30] Prüss-Üstün A., Mathers C., Corvalán C., Woodward A. (2003). Environmental Burden of Disease Series No. 1; Introduction and Methods: Assessing the Environmental Burden of Disease at National and Local Levels.

[31] (2015). Economic Cost of the Health Impact of Air Pollution in Europe: Clean Air, Health and Wealth.

[32] Istamto T., Houthuijs D., Lebret E. (2014). Willingness to Pay to Avoid Health Risks from Road-Traffic-Related Air Pollution and Noise across Five Countries. Sci. Total Environ..

[33] Istamto T., Houthuijs D., Lebret E. (2014). Multi-country willingness to pay study on road-traffic environmental health effects: Are people willing and able to provide a number?. Environ. Health.

[34] Fanelli D., Glänzel W. (2013). Bibliometric Evidence for a Hierarchy of the Sciences. PLoS ONE.

[35] Branches of Science. en.wikipedia.org/wiki/Branches_of_science.

[36] Boyack K.W., Klavans R., Börner K. (2005). Mapping the backbone of science. Scientometrics.

[37] Spruijt P. (2014). Roles of scientists as policy advisers on complex issues: A literature review. Environ. Sci. Policy.

[38] Cohen M.D., Levinthal D.A. (1990). Absorptive capacity: A new perspective on learning and innovation. Admin. Sci. Quart..

[39] Nooteboom B. (2000). Learning by Interaction: Absorptive Capacity, Cognitive Distance and Governance. J. Manag. Gov..

[40] Leonard-Barton D. (1995). Wellsprings of Knowledge: Building and Sustaining the Sources of Innovation.

[41] Grenelle_Environnement. https://en.wikipedia.org/wiki/Grenelle_Environnement.

[42] (2014). Explicitly Dealing with Safety’ (in Dutch) Bewust Omgaan Met Veiligheid, Rode Draden; Een Proeve van een IenM-Breed Afwegingskader Veiligheid.

[43] (2014). Convergence: Facilitating Transdisciplinary Integration of Life Sciences, Physical Sciences, Engineering, and Beyond.

